# Enhanced Positioning Algorithm of ARPS for Improving Accuracy and Expanding Service Coverage

**DOI:** 10.3390/s16081284

**Published:** 2016-08-12

**Authors:** Kyuman Lee, Hoki Baek, Jaesung Lim

**Affiliations:** 1 Department of Computer Engineering, Ajou University, 206 World cup-ro, Yeongtong-gu, Suwon 16499, Korea; mool717@ajou.ac.kr; 2 Department of Military Digital Convergence, Ajou University, 206 World cup-ro, Yeongtong-gu, Suwon 16499, Korea; neloyou@ajou.ac.kr

**Keywords:** accuracy enhancement, re-estimation, positioning algorithm, virtual measurement, dilution of precision

## Abstract

The airborne relay-based positioning system (ARPS), which employs the relaying of navigation signals, was proposed as an alternative positioning system. However, the ARPS has limitations, such as relatively large vertical error and service restrictions, because firstly, the user position is estimated based on airborne relays that are located in one direction, and secondly, the positioning is processed using only relayed navigation signals. In this paper, we propose an enhanced positioning algorithm to improve the performance of the ARPS. The main idea of the enhanced algorithm is the adaptable use of either virtual or direct measurements of reference stations in the calculation process based on the structural features of the ARPS. Unlike the existing two-step algorithm for airborne relay and user positioning, the enhanced algorithm is divided into two cases based on whether the required number of navigation signals for user positioning is met. In the first case, where the number of signals is greater than four, the user first estimates the positions of the airborne relays and its own initial position. Then, the user position is re-estimated by integrating a virtual measurement of a reference station that is calculated using the initial estimated user position and known reference positions. To prevent performance degradation, the re-estimation is performed after determining its requirement through comparing the expected position errors. If the navigation signals are insufficient, such as when the user is outside of airborne relay coverage, the user position is estimated by additionally using direct signal measurements of the reference stations in place of absent relayed signals. The simulation results demonstrate that a higher accuracy level can be achieved because the user position is estimated based on the measurements of airborne relays and a ground station. Furthermore, the service coverage is expanded by using direct measurements of reference stations for user positioning.

## 1. Introduction

With the proliferation of location-based services and industries, position information and time have become important resources. The global navigation satellite system (GNSS) is a position, navigation and timing (PNT) source and has been used in various areas [1,2]. However, the GNSS may not be available in shaded areas, such as in indoor and underground environments, and is vulnerable to signal interferences by the surrounding area because of the weak received signal strength. Thus, several studies have been conducted to develop alternative systems [3,4].

Over the past few decades, pseudolite-based positioning systems have been studied as an alternative PNT source. Pseudolite-based positioning systems are divided into two types depending on where the pseudolites are installed: ground- and airborne-based systems. A ground-based pseudolite system, in which pseudolites are installed on buildings and the peaks of mountains from where they provide navigation signals, has the advantages of higher power and greater flexibility than the GNSS. In addition, system construction and maintenance are relatively simple and inexpensive. However, because all components are located on the ground, this system can be influenced by the multipath effect, and the accuracy of vertical estimation is poor [5,6,7,8]. In contrast, an airborne-based pseudolite system is configured by installing pseudolites on airborne platforms, such as unmanned aerial vehicles (UAVs) and stratospheric airships. Consequently, the system can cover a larger area than a ground-based pseudolite system, and the multipath effect is decreased. However, the positions of airborne pseudolites must be monitored to achieve accurate service, and delays in monitoring degrade the system accuracy [9,10,11,12]. In both of these pseudolite-based positioning systems, the user position accuracy is not high because the pseudolites are located at biased positions on the same plane with respect to the user. Therefore, studies have been conducted to enhance the accuracy of these systems. In the studies by Tuohino and Sultana [13,14], pseudolites were positioned at various altitudes to guarantee a good dilution of precision (DOP) by varying the configuration dimensions. However, these previous studies were limited in that the assistance of the GNSS or monitoring is required to precisely estimate the positions of airborne pseudolites.

Recently, the airborne relay-based positioning system (ARPS) was proposed to overcome the limitations of conventional pseudolite-based positioning systems, such as the multipath effect and monitoring delay [15]. In the ARPS, ground reference stations transmit navigation signals, and airborne relays transfer these signals to a user. The user then sequentially estimates the positions of the airborne relays and its own position using the relayed signals. Owing to this two-step positioning procedure, the ARPS is not affected by monitoring delays, and it can benefit from the advantages of both ground- and airborne-based positioning systems by using the airborne relay. Therefore, the performance can be improved as compared to that of pseudolite-based positioning systems. However, the vertical error is still relatively large because the user position is determined based on the estimated positions of airborne relays. Furthermore, the positioning is restricted when relayed navigation signals are lacking or when the user is outside the range of some relay coverage.

To improve the accuracy and expand the coverage of the ARPS, we propose an enhanced positioning algorithm. The main idea of this enhanced algorithm is that the virtual and direct measurements of reference stations are adaptably used in the calculation process to reduce the effects of geometrical factors and to improve positioning possibility. The enhanced algorithm is designed such that the user re-estimates its own position after initial positioning by adding a virtual measurement of a reference station when there are more than four navigation signals for positioning. Otherwise, if the number of signals is insufficient, the direct measurements of nearby reference stations are used to derive the navigation equation for the user. The accuracy of the user can thus be improved relative to previous methods in environments that are not optimal because the user position is estimated based on the measurements of the ground reference station, as well as of the airborne relays. Moreover, positioning is possible over a wider area because a lack of navigation signals for user estimation is compensated by using direct signals received from nearby reference stations. We suggest that this enhanced algorithm will be beneficial for the relative navigation of the joint tactical information distribution system (JTIDS), which usually operates in battlefields where signal outage and the blockage of signals occur frequently.

The rest of this paper is organized as follows. In Section 2, the ARPS is briefly detailed, and the enhanced positioning algorithm is described in Section 3. The simulation construction and results are presented in Section 4. Finally, the paper is concluded in Section 5.

## 2. Airborne Relay-Based Regional Positioning System

**Figure 1:**
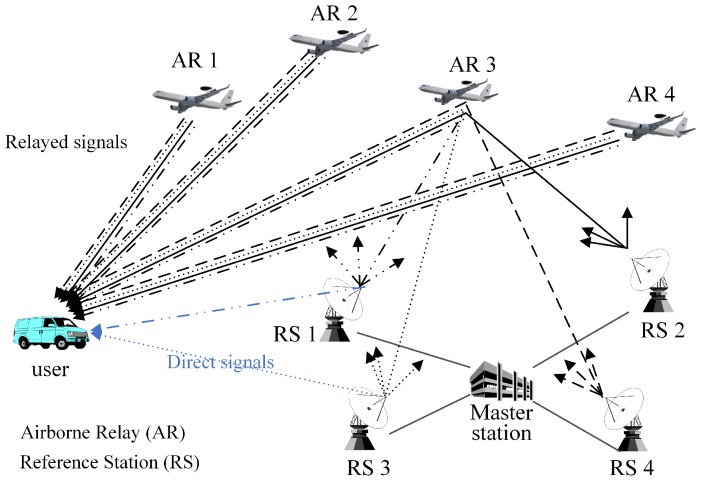
Configuration of the airborne relay-based positioning system.

The ARPS, which was proposed in order to address the problems of the pseudolite-based positioning system as a GNSS alternative regional positioning system, is comprised of a master station, reference stations, airborne relays and a user, as shown in Figure 1 [15].

The master station provides the absolute time reference of the system and generates correction information by using the navigation signals transferred from the airborne relays. The reference stations periodically transmit navigation signals based on their known positions and time similar to pseudolites and GNSS satellites. They are synchronized with each other with respect to the master station. Airborne relays are new components not found in the conventional pseudolite-based positioning system. These airborne relays transfer navigation signals back to the user via different frequencies as soon as they are received from the reference stations, like bent-pipe satellites. The user estimates the positions of the airborne relays and its own position using relayed navigation signals through a two-step procedure. In the first step, the positions of the airborne relays are determined based on the known positions of the reference stations and the time difference of arrival (TDOA) of the relayed signals. Then, the user position is determined using the estimated positions of airborne relays and the pseudoranges from each airborne relay to the user.

The ARPS can achieve the advantages of both ground-based and airborne-based positioning systems by employing airborne relays. Therefore, the multipath effect and near far problem, which are incurred in ground-based positioning systems, are not significant, and the coverage is large. Moreover, monitoring and measuring to estimate airborne pseudolite positions are not required, because the user can sequentially estimate the airborne relay positions and its own position. Thus, the mobility of airborne relays does not affect the accuracy of the system. However, despite the enhancement of performance as compared to that of the pseudolite-based positioning system, relatively large vertical errors occur as compared to horizontal errors, because the user position is estimated on the basis of the positions of the airborne relays, which are located toward the same direction. This geometric feature results in the major axis of the error ellipse being in the vertical direction. Furthermore, the user cannot estimate its position when it is not within an area covered by airborne relays, and the relayed navigation signals are insufficient, because the existing positioning algorithm is processed using only relayed signals.

## 3. Enhanced Positioning Algorithm for ARPS

### 3.1. Procedures of the Enhanced Algorithm

In the ARPS, which employs the relay concept for positioning, the user can receive both relayed navigation signals from airborne relays and direct navigation signals from ground reference stations. We propose an enhanced positioning algorithm that uses this structural characteristic in order to improve the vertical accuracy and expand the service coverage of ARPS.

**Figure 2:**
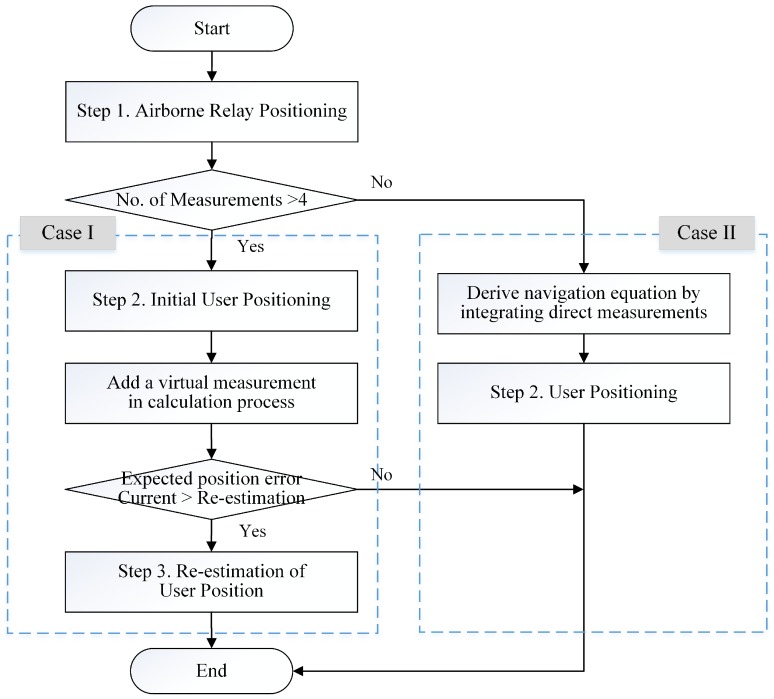
Positioning procedures of the enhanced algorithm.

The procedure of the enhanced algorithm is divided into two cases, as shown in Figure 2. The first case is where the required number of measurements for user positioning is met, and the second case is where it is not. The positioning procedure in Case I comprises three steps, because a re-estimation procedure is added for accuracy enhancement. The first and second steps are for estimating the positions of the airborne relays and the initial user location in a manner similar to that of the existing algorithm. After the initial position estimation of the user, the user re-estimates its own position in the third step. In this step, a virtual measurement of a reference station is added in the calculation process instead of a direct measurement, which is received directly from the reference station, because large errors could be included in the direct measurement caused by the multipath effect and tropospheric delay at a low elevation angle. The virtual measurement from the reference station to the user is calculated on the basis of the estimated initial position of the user and the position information of the reference station, which is included in the relayed signals. Therefore, we have named this measurement the virtual pseudorange, because reception of the navigation signal from the reference station is not required, and the measurement includes the error in the initial user position instead of errors associated with signal propagation. However, the geometry of the added reference station may exert an adverse effect if the airborne relays are already well distributed with respect to the user. Furthermore, although the DOP for re-estimation can be improved by additionally using the reference station in the estimation, the error in the user positioning may increase because of a virtual measurement error. Thus, the user determines whether to re-estimate its own position or not prior to the third step by comparing the expected position error in the second and third steps.

In contrast to that in Case I, the procedure in Case II consists of two steps. The airborne relay positions are estimated in the first step, as in the existing algorithm, whereas the second step is processed differently because of the lack of relayed signal measurements for user positioning. In the second step, the user derives a navigation equation to estimate its own position by adding the direct measurements of the reference station. However, the direct measurements include large errors, and it is difficult to guarantee a good DOP for positioning when the receiver is located in the periphery of the segment disposition. Therefore, the accuracy of the user positioning is lower than in Case I in the second step. Because the accuracy of the initial user positioning is degraded, the re-estimation of the user position is not performed in Case II.

### 3.2. Mathematical Expression of Positioning Procedures

The mathematical expressions of the enhanced algorithm are given in detail in this section. We have listed the important variables to represent the procedure in Table 1.

The ARPS user should estimate the positions of the airborne relays before determining its own position. In the enhanced algorithm, the positions of the airborne relays are estimated in the same manner as in the existing ARPS algorithm. Airborne relay positioning is performed using the TDOA of the relayed navigation signals transferred by each relay. The difference in the pseudoranges between the first/*j*-th reference stations and *i*-th airborne relay can be represented as [15]:

(1) $$\begin{array}{c} {}^{r_{j}}\nabla^{r_{1}}\rho_{a_{i}}=c\times \left(t_{r_{j},a_{i}}-t_{r_{1},a_{i}}\right),\;\;\;\;1\leq i\leq I,1\leq j\leq J\\ \;\;\;=e_{a_{i}}^{r_{j}}\cdot \left(R_{r_{j}}-R^{a_{i}}\right)-e_{a_{i}}^{r_{1}}\cdot \left(R_{r_{1}}-R^{a_{i}}\right){+}^{r_{j}}\nabla^{r_{1}}\tau_{a_{i}}{+}^{r_{j}}\nabla^{r_{1}}m_{a_{i}}{+}^{r_{j}}\nabla^{r_{1}}n_{a_{i}} \end{array}$$

where *c* denotes the speed of light and *τ*, *m* and *n* signify the tropospheric delay, multipath delay and thermal noise, respectively, that are included in the pseudorange.

With *J* reference stations, the navigation equation for the estimation of the *i*-th airborne relay is derived as Equation (2). The *i*-th airborne relay position is determined by the nonlinear least squares method using the Levenberg–Marquardt algorithm [16]. The user repeats the first step until the positions of all of the airborne relays are estimated.

(2) $$\begin{array}{c} \begin{array}{c} \left[\begin{array}{c} e_{a_{i}}^{r_{1}}-e_{a_{i}}^{r_{2}}\\ e_{a_{i}}^{r_{1}}-e_{a_{i}}^{r_{3}}\\ \vdots \\ e_{a_{i}}^{r_{1}}-e_{a_{i}}^{r_{J}} \end{array}\right] \end{array}\;\begin{array}{c} \cdot R^{a_{i}}= \end{array}\;\begin{array}{c} \left[\begin{array}{c} {}^{r_{2}}\nabla^{r_{1}}\rho_{a_{i}}-e_{a_{i}}^{r_{2}}\cdot R_{r_{2}}+e_{a_{i}}^{r_{1}}\cdot R_{r_{1}}{-}^{r_{2}}\nabla^{r_{1}}\tau_{a_{i}}{-}^{r_{2}}\nabla^{r_{1}}m_{a_{i}}{-}^{r_{2}}\nabla^{r_{1}}n_{a_{i}}\\ {}^{r_{3}}\nabla^{r_{1}}\rho_{a_{i}}-e_{a_{i}}^{r_{3}}\cdot R_{r_{3}}+e_{a_{i}}^{r_{1}}\cdot R_{r_{1}}{-}^{r_{3}}\nabla^{r_{1}}\tau_{a_{i}}{-}^{r_{3}}\nabla^{r_{1}}m_{a_{i}}{-}^{r_{3}}\nabla^{r_{1}}n_{a_{i}}\\ \vdots \\ {}^{r_{J}}\nabla^{r_{1}}\rho_{a_{i}}-e_{a_{i}}^{r_{J}}\cdot R_{r_{J}}+e_{a_{i}}^{r_{1}}\cdot R_{r_{1}}{-}^{r_{J}}\nabla^{r_{1}}\tau_{a_{i}}{-}^{r_{J}}\nabla^{r_{1}}m_{a_{i}}{-}^{r_{J}}\nabla^{r_{1}}n_{a_{i}} \end{array}\right] \end{array} \end{array}$$

**Figure 3:**
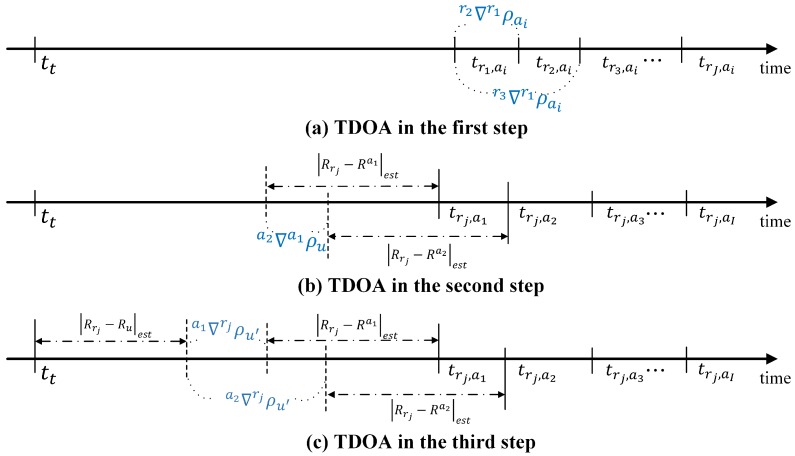
Time difference of arrival for each step.

After the position estimation of the airborne relays, the user determines its own initial position. The existing ARPS algorithm uses the time of arrival (TOA) of the relayed signals in the second step; in contrast, the TDOA of the relayed signals is used to provide the consistency of positioning procedures in the enhanced algorithm. The difference in the TDOAs of each step is shown in Figure 3.

The ARPS user receives multiple relayed signals because airborne relays transfer all navigation signals received from each reference station. However, each relayed signal has different measurement errors according to the location of each reference station. Therefore, the accuracy of the initial user position would be affected depending on which relayed signals are used in the TDOA calculation. Consequently, the pseudorange differences between airborne relays and the user corresponding to each reference station are averaged to mitigate the influence of geometrical factors. The average pseudorange difference between the first/*i*-th airborne relays and the user is derived as:

(3) $$\begin{array}{c} {}^{a_{i}}\nabla^{a_{1}}{\hat{\rho }}_{u}=\frac{1}{J}\sum_{k=1}^{J}\left[c\times \left(t_{r_{k},a_{i}}-t_{r_{k},a_{1}}\right)-|R_{r_{k}}-R^{a_{i}}{|}_{est}+{\left|R_{r_{k}}-R^{a_{1}}\right|}_{est}\right]\\ \;\;\;\;=e_{u}^{a_{i}}\cdot \left(R^{a_{i}}-R_{u}\right)-e_{u}^{a_{1}}\cdot \left(R^{a_{1}}-R_{u}\right)+{\hat{\tau }}_{u}^{a_{i}}+{\hat{m}}_{u}^{a_{i}}+{\hat{n}}_{u}^{a_{i}}-{\hat{\tau }}_{u}^{a_{1}}-{\hat{m}}_{u}^{a_{1}}-{\hat{n}}_{u}^{a_{1}} \end{array}$$

where $\hat{\tau }$, $\hat{m}$ and $\hat{n}$ indicate the averaged tropospheric delay, multipath delay and thermal noise, respectively, which are included in the averaged pseudorange. Using Equation (3), the following set of equations for *I* airborne relays is derived. Consequently, the user can obtain its initial position by the nonlinear least squares method, as in the first step.

(4) $$\begin{array}{c} \begin{array}{c} \left[\begin{array}{c} e_{u}^{a_{1}}-e_{u}^{a_{2}}\\ e_{u}^{a_{1}}-e_{u}^{a_{3}}\\ \vdots \\ e_{u}^{a_{1}}-e_{u}^{a_{I}} \end{array}\right] \end{array}\;\begin{array}{c} \cdot R_{u}= \end{array}\;\begin{array}{c} \left[\begin{array}{c} {}^{a_{2}}\nabla^{a_{1}}{\hat{\rho }}_{u}-e_{u}^{a_{2}}\cdot R^{a_{2}}+e_{u}^{a_{1}}\cdot R^{a_{1}}{-}^{a_{2}}\nabla^{a_{1}}{\hat{\tau }}_{u}{-}^{a_{2}}\nabla^{a_{1}}{\hat{m}}_{u}{-}^{a_{2}}\nabla^{a_{1}}{\hat{n}}_{u}\\ {}^{a_{3}}\nabla^{a_{1}}{\hat{\rho }}_{u}-e_{u}^{a_{3}}\cdot R^{a_{3}}+e_{u}^{a_{1}}\cdot R^{a_{1}}{-}^{a_{3}}\nabla^{a_{1}}{\hat{\tau }}_{u}{-}^{a_{3}}\nabla^{a_{1}}{\hat{m}}_{u}{-}^{a_{3}}\nabla^{a_{1}}{\hat{n}}_{u}\\ \vdots \\ {}^{a_{I}}\nabla^{a_{1}}{\hat{\rho }}_{u}-e_{u}^{a_{I}}\cdot R^{a_{I}}+e_{u}^{a_{1}}\cdot R^{a_{1}}{-}^{a_{I}}\nabla^{a_{1}}{\hat{\tau }}_{u}{-}^{a_{I}}\nabla^{a_{1}}{\hat{m}}_{u}{-}^{a_{I}}\nabla^{a_{1}}{\hat{n}}_{u} \end{array}\right] \end{array} \end{array}$$

In Case I, the user re-estimates its position using the virtual pseudorange measurement from the reference stations to the user, which is generated by using the reference station information in the relayed signals and the estimated initial position of the user, in order to enhance the user accuracy. The TDOA in which the virtual measurement is integrated is represented as:

(5) $$\begin{array}{c} {}^{a_{i}}\nabla^{r_{j}}{\hat{\rho }}_{u^{\prime }}=\frac{1}{J}\sum_{k=1}^{J}\left[c\times \left(t_{r_{k},a_{i}}-t_{t}\right)-{\left|R_{r_{k}}-R^{a_{i}}\right|}_{est}\right]-{\left|R_{r_{j}}-R_{u}\right|}_{est}\\ \;\;\;\;=e_{u^{\prime }}^{a_{i}}\cdot \left(R^{a_{i}}-R_{u^{\prime }}\right)-e_{u^{\prime }}^{r_{j}}\cdot \left(R_{r_{j}}-R_{u^{\prime }}\right)+{\hat{\tau }}_{u}^{a_{i}}+{\hat{m}}_{u}^{a_{i}}+{\hat{n}}_{u}^{a_{i}}-\epsilon_{u}^{r_{j}} \end{array}$$

where $t_{t}$ denotes the navigation signal transmission time of all reference stations. For example, if $t_{t}=2$, all reference stations broadcast navigation signals at two seconds in the system time. $\epsilon_{u}^{r_{j}}$ indicates the virtual measurement error caused by the initial positioning error of the user. From Equation (5), the re-estimation equation of the third step with the measurements of *L* reference stations and *I* airborne relays is derived as follows, and the final user position can be resolved with the nonlinear least squares method using the Levenberg-Marquardt algorithm.

(6) $$\begin{array}{c} \begin{array}{c} \left[\begin{array}{c} e_{u^{\prime }}^{r_{1}}-e_{u^{\prime }}^{r_{2}}\\ \vdots \\ e_{u^{\prime }}^{r_{1}}-e_{u^{\prime }}^{r_{L}}\\ e_{u^{\prime }}^{r_{1}}-e_{u^{\prime }}^{a_{1}}\\ \vdots \\ e_{u^{\prime }}^{r_{1}}-e_{u^{\prime }}^{a_{I}} \end{array}\right] \end{array}\;\begin{array}{c} \cdot R_{u^{\prime }}= \end{array}\;\begin{array}{c} \left[\begin{array}{c} {}^{r_{2}}\nabla^{r_{1}}\rho_{u^{\prime }}-e_{u^{\prime }}^{r_{2}}\cdot R_{r_{2}}+e_{u^{\prime }}^{r_{1}}\cdot R_{r_{1}}-\epsilon_{u}^{r_{2}}+\epsilon_{u}^{r_{1}}\\ \vdots \\ {}^{r_{L}}\nabla^{r_{1}}\rho_{u^{\prime }}-e_{u^{\prime }}^{r_{L}}\cdot R_{r_{L}}+e_{u^{\prime }}^{r_{1}}\cdot R_{r_{1}}-\epsilon_{u}^{r_{L}}+\epsilon_{u}^{r_{1}}\\ {}^{a_{1}}\nabla^{r_{1}}{\hat{\rho }}_{u^{\prime }}-e_{u^{\prime }}^{a_{1}}\cdot R^{a_{1}}+e_{u^{\prime }}^{r_{1}}\cdot R_{r_{1}}-{\hat{\tau }}_{u}^{a_{1}}-{\hat{m}}_{u}^{a_{1}}-{\hat{n}}_{u}^{a_{1}}+\epsilon_{u}^{r_{1}}\\ \vdots \\ {}^{a_{I}}\nabla^{r_{1}}{\hat{\rho }}_{u^{\prime }}-e_{u^{\prime }}^{a_{I}}\cdot R^{a_{I}}+e_{u^{\prime }}^{r_{1}}\cdot R_{r_{1}}-{\hat{\tau }}_{u}^{a_{I}}-{\hat{m}}_{u}^{a_{I}}-{\hat{n}}_{u}^{a_{I}}+\epsilon_{u}^{r_{1}} \end{array}\right] \end{array} \end{array}$$

However, the accuracy of the user may be degraded by the error in the virtual measurement and the geometry of the added reference station in the third step. Therefore, the user calculates the expected position errors based on the added reference station by multiplying the DOP of the airborne relays and the reference station with respect to the user by the user range error (URE), and the minimum of the expected position errors is compared to the expected position error of the initial estimation to determine whether to re-estimate. The URE has been analyzed by Mc-Graw in clean environments as a function of the elevation angle [17]. The expected position error and DOP are defined as [18]:

(7) $$RMS\left(expected\;\;position\;\;error\right)=\sigma_{ure}\times DOP$$

(8) $$HDOP=\sqrt{H_{11}+H_{22}},\;VDOP=\sqrt{H_{33}},\;PDOP=\sqrt{H_{11}+H_{22}+H_{33}}$$

where $\sigma_{ure}$ denotes the URE and *H* is a covariance matrix given by:

(9) $$\begin{array}{c} H={\left(G^{T}G\right)}^{-1},\;G=\left[\begin{array}{cccc} \left(x_{u}-x_{a_{1}}\right)/\rho_{u}^{a_{1}} & \left(y_{u}-y_{a_{1}}\right)/\rho_{u}^{a_{1}} & \left(z_{u}-z_{a_{1}}\right)/\rho_{u}^{a_{1}} & 1\\ \vdots & \vdots & \vdots & \vdots \\ \left(x_{u}-x_{a_{I}}\right)/\rho_{u}^{a_{I}} & \left(y_{u}-y_{a_{I}}\right)/\rho_{u}^{a_{I}} & \left(z_{u}-z_{a_{I}}\right)/\rho_{u}^{a_{I}} & 1\\ \left(x_{u}-x_{r_{1}}\right)/\rho_{u}^{r_{1}} & \left(y_{u}-y_{r_{1}}\right)/\rho_{u}^{r_{1}} & \left(z_{u}-z_{r_{1}}\right)/\rho_{u}^{r_{1}} & 1\\ \vdots & \vdots & \vdots & \vdots \\ \left(x_{u}-x_{r_{L}}\right)/\rho_{u}^{r_{L}} & \left(y_{u}-y_{r_{L}}\right)/\rho_{u}^{r_{L}} & \left(z_{u}-z_{r_{L}}\right)/\rho_{u}^{r_{L}} & 1 \end{array}\right] \end{array}$$

where *G* refers to the geometry matrix and *x*, *y* and *z* denote the position of the user, airborne relays and reference stations in three dimensions.

As stated in Section 3.1, when the number of measurements for user positioning is insufficient, the procedures are processed differently. In this case, the direct measurements of the reference station near the user are used for deriving the navigation equation of the user in the second step. The TDOA of the relayed measurement based on the direct measurement, which is first received, is derived as:

(10) $$\begin{array}{c} {}^{a_{i}}\nabla^{r_{j}}{\hat{\rho }}_{u}=\frac{1}{J}\sum_{k=1}^{J}\left[c\times t_{r_{k},a_{i}}-{\left|R_{r_{k}}-R^{a_{i}}\right|}_{est}\right]-c\times t_{r_{j}}\\ \;\;\;\;=e_{u}^{a_{i}}\cdot \left(R^{a_{i}}-R_{u}\right)-e_{u}^{r_{j}}\cdot \left(R_{r_{j}}-R_{u}\right)+{\hat{\tau }}_{u}^{a_{i}}+{\hat{m}}_{u}^{a_{i}}+{\hat{n}}_{u}^{a_{i}}-\tau_{u}^{r_{j}}-m_{u}^{r_{j}}-n_{u}^{r_{j}} \end{array}$$

Using Equation (10), the navigation equation for estimating the user position in Case II can be constructed in a manner similar to that in Case I.

## 4. Simulation Results and Discussion

### 4.1. Simulation Assumptions and Construction

#### 4.1.1. Simulation Assumptions

In the ARPS, the user must receive more than four relayed navigation signals from each airborne relay because a minimum of four navigation signals are required to estimate the three-dimensional position of the airborne relay based on the TDOA [19]. We assumed that the line of sight of all airborne relays is guaranteed for each reference station. Therefore, the user is considered to receive all of the reference station signals from the airborne relays.

The existing algorithm of the ARPS uses only relayed navigation signals. In contrast, the enhanced algorithm uses direct navigation signals, which are received directly from the near reference stations, as well as relayed navigation signals in order to enhance the user performance. Thus, we assumed that the user can receive both signals by modifying the receiver structure or the multiple access control scheme.

The ARPS positioning algorithm may not be affected by synchronization error if the time synchronization among reference stations is maintained within 20 ns [15]. As discussed in [6], nanosecond accuracy can be achieved through wireless synchronization. Thus, we did not consider the effect of the time offset among reference stations. Furthermore, the time offset of the user is negligible, because this can be removed by differencing the pseudorange measurement in the positioning procedures.

#### 4.1.2. Simulation Model Construction

**Figure 4:**
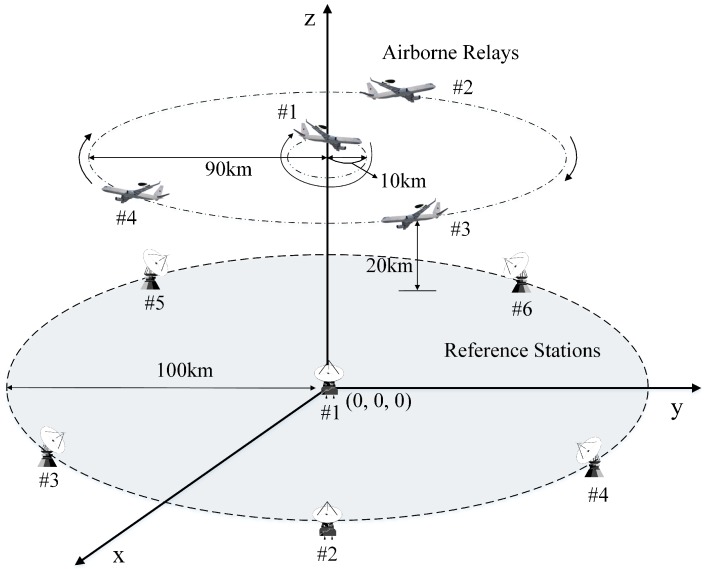
Segment disposition for simulations.

The physical location of system segments affects the accuracy in the navigation systems. We defined the disposition of segments with the consideration of practical environments, as shown in Figure 4.

The altitude of airborne relays was set at 20 km considering the UAV and stratospheric airship altitudes. The user must receive at least four signals from the airborne relays to calculate its position. Therefore, the radius of the airborne relay trajectory was set to 90 km in order to ensure an overlap of each airborne relay coverage, because the coverage, which is the radius of the visible region on the ground with an elevation angle of 5${}^{\circ }$, is 195 km at an altitude of 20 km. We assumed that the airborne relays are rotated along a circular orbit while maintaining a predetermined distance from each other. One of them is rotated relative to the center of a circle with a 10-km radius for a good DOP. Furthermore, we set the radius of the disposition of the reference stations to 100 km to guarantee that the estimation results of the airborne relays are good. These reference stations were distributed in the same manner as the airborne relays, but the locations were fixed. The master station was not considered in the simulations because it does not influence positioning performance. The number of airborne relays and reference stations was set to four and six, respectively, as in [15], and the speed of the airborne relays was set to 150 m/s. Moreover, the position change in the airborne relays, which results from the propagation time difference of the navigation signals during the relay, is considered.

#### 4.1.3. Measurement Errors

The ARPS discussed in this study is based on code-phased pseudorange measurements. The measurements are corrupted by several sources of error, which are related to the propagation medium and receiver. Herein, we consider three factors that contribute to airborne relay and user positioning measurement errors: the multipath effect, tropospheric delay and receiver noise.

The Hopfield model, which is stable for all ranges of elevation angles, is used to determine the tropospheric error [20]. The tropospheric delay error consists of dry and wet components ($m\;=\;d_{dry}+\;d_{wet}$) and is defined as:

(11) $$d_{*}=\frac{10^{-6}}{5}\times N_{*}\times D\times \left({\left(1-\frac{h_{rx}}{h_{*}}\right)}^{5}-{\left(1-\frac{h_{tx}}{h_{*}}\right)}^{5}\right)\times \frac{h_{*}}{h_{rx}-h_{tx}},*\in \left\{dry,wet\right\}$$

(12) $$\begin{array}{c} N_{dry}=77.6\times \frac{P}{T},\;N_{wet}=22770\times \frac{f}{T^{2}}\times 10^{\frac{7.4475\times (T-273)}{T-38.3}} \end{array}$$

where $N_{*}$ denotes the refractive index defined by Equation (12), *D* is the slope distance between the transmitter and receiver of the navigation signal, $h_{rx}$ and $h_{tx}$ respectively represent the receiver and transmitter heights, $h_{*}$ is the fixed scaled height for the model ($h_{dry}=42.7$ km, $h_{wet}=12$ km) and *P*, *T* and *f* represent the atmospheric pressure, temperature and relative humidity, respectively (P=1010.25 mbar, T=291.15 K, f=50%).

In addition, the multipath error is provided using the satellite navigation (SatNav) toolbox, a collection of MATLAB code files for total system simulation [21]. The function for multipath error in the SatNav toolbox generates zero-elevation angle equivalent pseudorange multipath errors with a linear autoregressive model, which is characterized by a standard deviation of approximately 1.6 m and a time constant of approximately 2 min. Before these errors are applied to pseudorange measurements, they are scaled by the factor $M(=1-\frac{tan^{-1}\alpha }{tan^{-1}\left(\pi /2\right)})$ where *α* is the elevation angle between the transmitter and receiver of the navigation signal [22]. Finally, the error due to receiver noise is generated by multiplying the standard deviation corresponding to the respective signal type by a normal distributed random number (n∼N(0,1)) [23].

### 4.2. Simulation Results and Discussion

To evaluate the performance of the enhanced algorithm, we performed simulations using MATLAB. First, we analyzed the re-estimation accuracy according to the measurement of the reference station, and second, we conducted simulations for finding the optimum condition of the virtual measurement in the re-estimation procedure. Third, we compared the performances of the existing algorithm and the enhanced algorithm in the service area. Finally, to demonstrate the performance of the enhanced algorithm for Case II, we evaluated the accuracy of the user positioning according to the distance from the center of the segment disposition.

#### 4.2.1. Influence of Measurement on Re-Estimation

In the enhanced algorithm, both the direct and virtual measurements of reference stations can be used for re-estimation. The first simulation was conducted to confirm the effect on accuracy according to the measurement, and we compared the re-estimation results when a single measurement was added.

**Figure 5:**
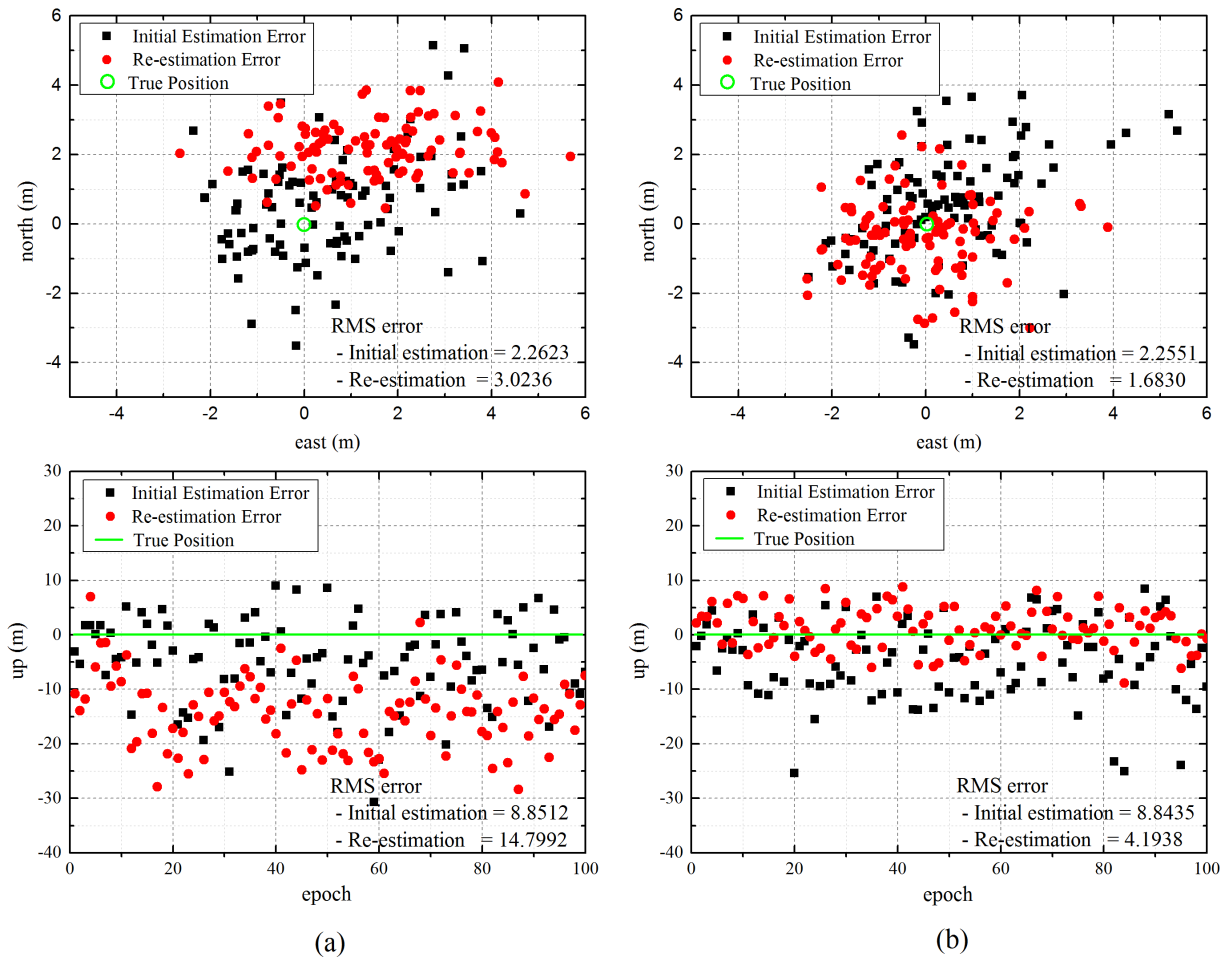
Re-estimation errors of the user for direct and virtual measurements. (**a**) Results for direct measurement; (**b**) Results for virtual measurement.

Figure 5 represents the east, north and up (ENU) errors of the user located at (40,000, 50,000, 50) when the user re-estimates its own position by adding the direct measurement or virtual measurement of the reference station. As expected, the re-estimation results for the direct measurement show that the user position error is increased, in particular in the vertical aspect, despite the improvement in the position DOP (PDOP) from 6.4011 to 3.0448, because of the measurement errors caused by the multipath and tropospheric delay, whereas the up error of the virtual measurement results decreases to 4.6497 m as compared to the existing algorithm. This is because the effect of measurement errors, which are large at a low elevation angle, is reduced by using a virtual measurement instead of a direct signal measurement, although inaccuracy is still caused by the error in the initial position of the user. Thus, the enhanced algorithm was designed such that the virtual measurement is used only for re-estimation.

#### 4.2.2. Relationship between Accuracy and Geometry

As with the regional positioning system, the accuracy of the ARPS depends on the geometry of the reference stations, airborne relays and the user. In the second simulation, we analyzed the re-estimation performance while changing the geometry, depending on the number of virtual measurements and the reference station positions, which are added to the navigation equation of the user in the re-estimation, in order to find the optimum condition. To evaluate different geometries, the user positions were set to (10,000, 10,000, 10) and (60,000, 60,000, 10) for good and poor geometries, respectively.

**Figure 6:**
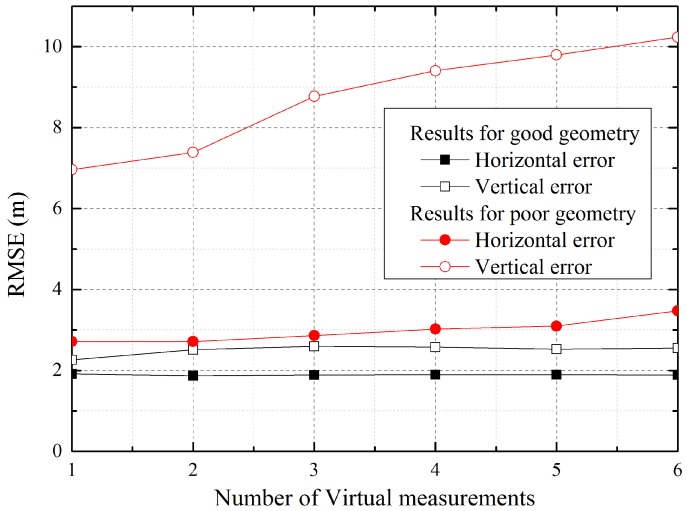
User accuracy according to the number of virtual measurements.

Figure 6 shows the horizontal and vertical root mean square error (RMSE) of the user for good and poor geometries. As shown in the graph, the user accuracy with poor geometry is degraded with an increase in the number of virtual measurements due to the error included in the initial user positioning, although the change in the horizontal error is small because the influence on the horizontal aspect exerted by the re-estimation based on the measurements of the various altitude platforms is not large. However, the results for good geometry are rarely affected by the number of virtual measurements, because the initial positioning accuracy, which influences the re-estimation accuracy, is more precise when the reference stations and airborne relays are already well distributed toward the user. Based on these results, it is demonstrated that more than one measurement is not required. Therefore, one virtual measurement was used for re-estimation in the remaining simulations.

The location of the reference station used to calculate the virtual measurement, which is added for re-estimation, is as important as the number of virtual measurements. Table 2 represents the PDOP of the re-estimation, where each reference station is applied after aligning the reference stations according to the distance from the initial user position. The PDOP is the value that indicates the three-dimensional accuracy of the user corresponding to the geometries of the airborne relays and the reference station of the virtual measurement; in general, a low value indicates a high accuracy. The results show that the accuracy is higher when the reference measurement adjacent to the initial user position is used for re-estimation. This is because a reference station that is located at a distance from the user worsens the geometry, since the position is biased to one direction. However, the influence on the geometry by the added reference station of the virtual measurement is small in the good geometry, where the airborne relays are uniformly distributed with respect to the user.

#### 4.2.3. Performance Comparison between Existing and Enhanced Algorithms

In this section, we describe the simulations, which were conducted to compare the existing algorithm and enhanced algorithm performances. For a fair simulation, the same segment disposition and measurement models were used, and the user position error was calculated by averaging the errors generated by the changes in geometry according to the movement of airborne relays.

**Figure 7:**
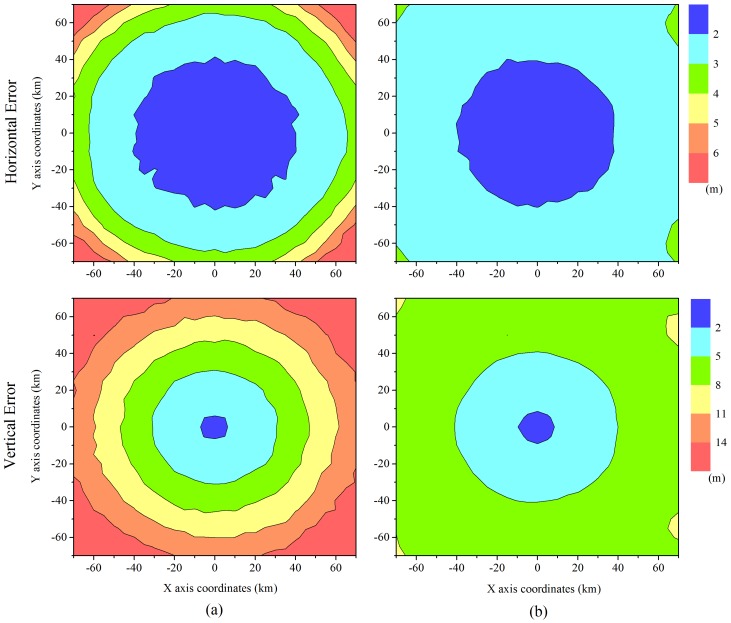
Horizontal and vertical accuracies of the user in a service area. (**a**) Existing algorithm; (**b**) Enhanced algorithm.

Figure 7 illustrates the horizontal and vertical error distributions of the user in a service area of 140 km × 140 km. The color represents the user error. As shown in the results, the enhanced algorithm provides a better accuracy over a wide area. According to Dai, the best configuration for a good DOP is that in which most of the segments are well distributed around a receiver and one of them is located on the zenith of the receiver [24]. Applying a virtual measurement of a ground reference station to the re-estimation renders the geometry for re-estimation similar to a good DOP configuration in the inverse direction. This results in the mitigation of the error ellipse forming in the vertical direction, and the accuracy can thus be improved. In the enhanced algorithm, whether to perform a re-estimation or not is determined for preventing an increase in the position error. Thus, there is no large enhancement in the center of the segment disposition, because the re-estimation is not conducted in the vicinity of the center, where the DOP is already good and a high accuracy level is guaranteed.

#### 4.2.4. Performance Evaluation for Case II

When the received navigation signals for user positioning are insufficient, the enhanced algorithm uses the measurement of direct signals, which are received from the reference stations. However, the direct signal measurements include large errors, which result from the multipath effect and tropospheric delay, and the user positioning accuracy is affected by the geometry of the reference stations of the added direct signals. Thus, we evaluated the performance of the user for Case II. In the simulations, we assumed that the user received three relayed navigation signals from near airborne relays. Therefore, the number of added direct signal measurements for user positioning is one, considering the minimum positioning requirement, and it is received from the nearest reference station.

**Figure 8:**
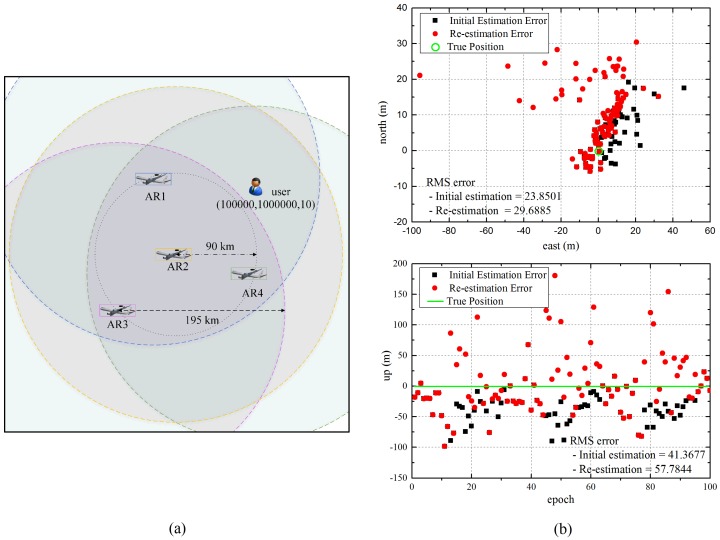
East, north and up (ENU) errors of the user positioning outside a coverage area. (**a**) User position in the simulation; (**b**) ENU errors of a user.

Figure 8 shows the ENU errors of the user located at (100,000, 100,000, 10), where it is outside the coverage of an airborne relay, and the effect of re-estimation on the accuracy is analyzed. The results demonstrate that large errors occurred in the user positioning as compared to the results for Case I, which are estimated based on airborne relays. This is because the user position is estimated using the measurements of airborne relays and a reference station that are close together, and the measurement error of the direct signals is large. Furthermore, the re-estimation error is increased. The re-estimation is performed using a virtual measurement, which is calculated based on the estimated initial user position in the second step. Therefore, the re-estimation error is amplified by the large initial error in the user positioning in Case II. For this reason, the user does not re-estimate its position in Case II.

**Figure 9:**
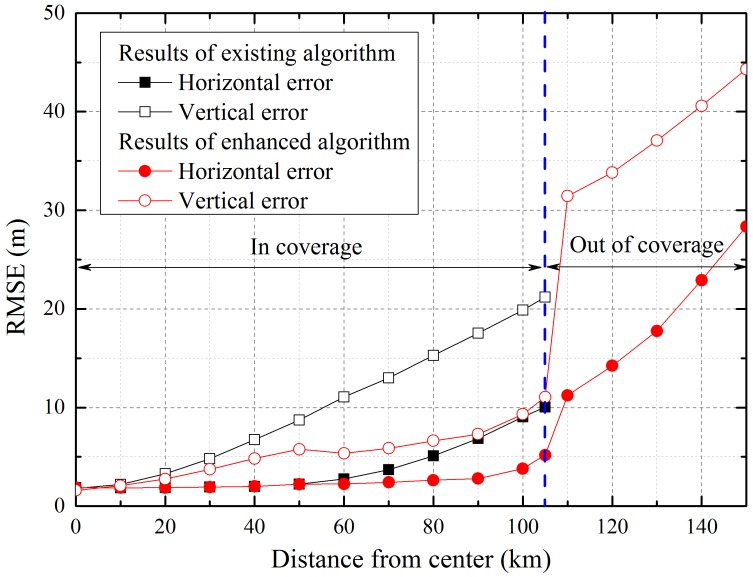
Horizontal and vertical accuracy according to the distance from the center.

Figure 9 shows a comparison of the user positioning accuracy according to the distance from the center of the segments’ disposition. In this simulation, we deemed that the user cannot receive relayed signals from one of the airborne relays because of the coverage restriction if the user is at a distance greater than 105 km from the center. As confirmed by the previous results, the enhanced algorithm improves the accuracy of user. Moreover, when the user is outside the coverage area of some airborne relays, positioning is available by integrating the direct measurements of the reference stations, although the error is increased because of poor geometry and measurement errors. In contrast, the existing algorithm, which uses only the relayed signals for positioning, cannot estimate the user position in areas not covered by the airborne relay.

## 5. Conclusions

The ARPS resolves the problems of pseudolite-based positioning systems, such as poor vertical observability, multipath effect and monitoring delay, by employing a navigation signal relay and a two-step positioning algorithm. However, the limitations of the conventional ARPS method include the relatively large vertical error and the restriction of positioning when relayed signals are blocked and absent. The causes of these limitations are that the user position is estimated based on airborne relays that are located in one direction with respect to the user and that the positioning is processed using only relayed signals. To enhance the user accuracy and positioning possibility, we have proposed an enhanced positioning algorithm. In the enhanced algorithm, the user re-estimates its own position using virtual measurements, as well as the relayed signal measurements. The re-estimation accuracy may be degraded because of a virtual measurement error and the influence of the geometry of the reference station. Therefore, the user compares the expected position error by multiplying the DOP by the URE before re-estimation in order to decide whether to re-estimate or not. Furthermore, if the navigation signals are insufficient, the user derives a navigation equation for user positioning by adding the direct signal measurements of near reference stations.

To evaluate the enhanced algorithm, various simulations were performed using MATLAB. The simulation results confirmed that the vertical accuracy is improved, and a relatively uniform accuracy is provided in the service area by diversifying the reference points of positioning. Moreover, a user that does not receive sufficient relayed navigation signals can estimate its own position. Finally, the enhanced algorithm can improve the accuracy and expand the service area of the system without the additional configuration of segments and augmentation data using structural characteristics.

## Figures and Tables

**Table 1:** Main notations for the mathematical expressions.

Variable	Description
$e_{a_{i}}^{r_{j}}$	Unit vector from the *i*-th airborne relay to the *j*-th reference station
$e_{u}^{a_{i}}$	Unit vector from the *i*-th airborne relay to the user
$R^{a_{i}}$	Position vector of the *i*-th airborne relay
$R_{r_{j}}$	Position vector of the *j*-th reference station
$R_{u}$	Initial user position vector
$R_{u^{\prime }}$	Final user position vector
$t_{r_{j},a_{i}}$	Measured reception time of the relayed signal by the *i*-th airborne relay from the *j*-th reference station to the user

$t_{r_{j}}$	Measured reception time of direct signal from the *j*-th reference station to the user
$\rho_{u}^{r_{j}}$	Measured pseudorange between the *j*-th reference station and the user
$\rho_{u}^{a_{i}}$	Estimated pseudorange between the *i*-th airborne relay and the user
$|R_{r_{k}}-R^{a_{i}}{|}_{est}$	Estimated distance between the *k*-th reference station and the *i*-th airborne relay
$|R_{r_{j}}-R_{u}{|}_{est}$	Estimated virtual pseudorange between the *j*-th reference station and the estimated initial user


**Table 2:** PDOP in accordance with the position of the reference station.

Distance from a User	Near <– – – – – – – – – – – – – – – – – –> Far					
**Good Geometry**	2.1087	2.2264	2.2565	2.2451	2.2304	2.2105
(10,000, 10,000, 10)						
**Poor Geometry**	4.8424	4.9252	5.6364	5.6731	5.7758	5.9863
(60,000, 60,000, 10)						
